# A comparison of eating disorder symptomatology, psychological distress and psychosocial function between early, typical and later onset anorexia nervosa

**DOI:** 10.1186/s40337-020-00337-w

**Published:** 2020-11-04

**Authors:** Zoe M. Jenkins, Lior M. Chait, Leonardo Cistullo, David J. Castle

**Affiliations:** 1 Mental Health Service, St. Vincent’s Hospital, Melbourne, Australia; 2 Department of Psychiatry, University of Melbourne, Melbourne, Australia

**Keywords:** Anorexia nervosa, Age of onset, Eating disorders, Depression, Anxiety, Stress, Psychosocial

## Abstract

**Objective:**

Epidemiological studies suggest that the incidence of anorexia nervosa (AN) is increasing in younger populations, with some evidence that clinical differences occur according to age of onset (AOO), which may impact prognostic outcomes. The current study sought to compare eating disorder (ED) symptomatology, psychological distress and psychosocial function between early onset (EO), typical onset (TO) and later onset (LO) AN in a large sample of treatment-seeking patients with a diagnosis of AN.

**Methods:**

Participants included 249 individuals with a diagnosis of AN who were assessed at an outpatient ED service. The sample was divided into three groups based on AOO; those with an AOO ≤14 years (*N* = 58) were termed ‘EO-AN’, those with an AOO between 15 and 18 years (*N* = 113) were termed ‘TO-AN’ and those with an AOO of > 18 years (*N* = 78) were termed ‘LO-AN’. Comparisons were made between AOO groups on assessments of ED symptomatology, psychological distress and psychosocial function.

**Results:**

EO-AN patients reported a significantly longer illness duration than both TO-AN and LO-AN groups. After controlling for effect of illness duration, the EO-AN group demonstrated significantly higher ED symptomatology and dysmorphic concern compared to the LO-AN group. The EO-AN group demonstrated significantly decreased cognitive flexibility compared to both the TO-AN and LO-AN groups.

**Discussion:**

These findings suggest that clinical differences do occur according to AOO in AN whereby EO-AN may represent a more severe form of illness that is not attributable to increased illness duration. Treatment strategies which specifically address patients with EO-AN may improve long term health outcomes and recovery.

## Plain English summary

Presentations of anorexia nervosa (AN) are increasing in young populations. There is some evidence that there are differences in clinical features according to age of onset. The current study compared individuals with early, typical and later onset AN on eating disorder (ED) symptoms, psychological distress and psychosocial function in a large sample of treatment-seeking patients. Individuals with an early onset AN demonstrated a longer illness duration, higher ED symptoms and dysmorphic concern than those with later onset AN. Moreover, individuals with early onset AN demonstrated decreased cognitive flexibility compared to both typical onset and later onset AN. Treatment strategies for individuals with early onset AN should incorporate environmental and developmental factors that may contribute to the development and maintenance of AN.

## Introduction

Anorexia nervosa (AN) is a severe illness that has an approximate lifetime prevalence of 1.7% [1], is associated with significant psychiatric comorbidity [2], and demonstrates the highest mortality rate of any psychiatric disorder [3]. The c is between 15 and 19 years old [4] and while population-based studies suggest that the overall incidence of AN in the general population has remained static since the 1970’s [5, 6], there has been a proportional increase in childhood presentations (aged below 14 years) [7, 8] and a trend for individuals presenting with AN at a younger age [9, 10] over the past two decades. AOO has been demonstrated as a clinically significant feature in various psychiatric illnesses including affective disorders [11, 12], psychotic disorders [13] and anxiety disorders [14, 15] and may have important prognostic implications [16]. Accordingly, exploration of clinical features across AOO may allow demarcation of symptom presentation and inform treatment prognosis.

Previous investigations into AOO in AN are inconclusive, with conflicting evidence surrounding the impact of AOO on ED severity, prognostic implications and psychological profile. Evidence for increased ED severity and poorer prognostic outcomes in EO-AN (as compared to LO-AN) have been demonstrated including; more rapid weight loss [17, 18], poorer long-term outcomes of low body weight and psychiatric comorbidity [19], and a longer duration of illness [20]. However, other studies have found no difference in severity of weight loss between EO- and LO-AN [21], fewer cases of extremely low weight in those with EO-AN compared to LO-AN [22], and a positive association between low body mass index (BMI) and increased AOO [23]. Assessments of the impact of AOO on psychological profile in individuals with AN also provide contrasting evidence. Whereas one study reported better self-esteem in those with EO-AN [24], others have reported that individuals with EO-AN demonstrate higher maturity fear, impulsivity and asceticism (interpreted as greater character fragility) than those with LO-AN [25].

A contributing factor to the discordant evidence to date may be the lack of consensus for what age constitutes ‘EO’ and ‘LO’ AN. EO-AN has previously been depicted as an AOO of < 14 years in some studies [22, 24, 26], < 16 years [25] and < 25 years in others [21], while others still have used puberty and menarche to demarcate AOO groups [27].

To date, there is a paucity of studies that compare levels of psychological distress, such as depression and anxiety and psychosocial functioning across different AOO groups in AN. Moreover, the majority of investigations into AOO in ED populations have utilised inpatient samples or retrospective healthcare records, with fewer investigations into outcomes in community and out-patient ED groups.

The current study aims to compare clinical presentation across three AOO groups in individuals with a diagnosis of AN in a large sample of treatment-seeking adults at an out-patient ED service. Specifically, measures of ED symptomatology, psychological distress and psychosocial function will be compared across those with an EO-AN, typical-onset AN (TO-AN) and LO-AN groups. It is anticipated that those with EO-AN will exhibit increased ED symptomatology, psychological distress and more impaired psychosocial functioning than those with either TO-AN or LO-AN.

## Methods

### Participants and procedure

Data from all patients with a diagnosis of AN (*n* = 249) who were assessed for treatment at the Body Image and Eating Disorders Recovery Service (BETRS) at St. Vincent’s Hospital, Melbourne Australia between 2012 and 2019 were included in this study. The service provided at BETRS includes outpatient and day patient programs and is described elsewhere [28]. Diagnosis of AN was determined through a comprehensive assessment by specialist clinicians under the guidance of consultant psychiatrists in accordance with DSM-5 [29]. Data were collected upon initial presentation as part of a larger assessment protocol. The study was granted ethics approval from the Human Research Ethics Committee at St Vincent’s Hospital, Melbourne and all procedures were in line with the Declaration of Helsinki. Informed consent was obtained from all participants.

### Measures

Demographic and clinical information:

- Information relating to ethnicity, education, employment status and partnered status.
- Age of illness onset was self-reported by patients at initial assessment.
- The duration of illness was calculated as the duration from self-reported AOO until age at initial assessment.

Eating disorder symptomatology:

- BMI: Height and weight were assessed using calibrated instruments. The participant’s BMI was calculated by dividing their weight (kg) by the square of their height (m).
- The Eating Disorder Examination Questionnaire (EDE-Q) [30] is a 28-item self-report measure of psychological domains relevant to individuals with eating disorders. The questionnaire asks individuals to report on items in relation to the past 28 days and provides subscales of eating restraint, eating concern, weight concern, shape concern and global score, with higher scores on the EDE-Q indicate greater levels of disordered eating.
- Dysmorphic Concern: The Dysmorphic Concern Questionnaire (DCQ) is an assessment of levels of dysmorphic concern [31] and has gained support as a brief screening measure for BDD [32]. It is a self-report questionnaire that has 7 items rated on a 4-point Likert scale from 0 = “Not at all” to 3 = “Much more than most people”.
- AN subtype: During the assessment process and evaluation of ED diagnosis, clinicians at BETRS reported the AN subtype as either ‘restraint’ or ‘binge-purge’ as per DSM-5 criteria [29].

Psychological distress and psychosocial assessment:

- The Depression Anxiety Stress Scale (DASS-21) [33] is a 21-item self-report instrument designed to measure the three related negative emotional states of depression, anxiety and tension/stress over the past week. Individual subscale scores representing depression, anxiety and stress were used here.
- Self-efficacy: The General Self-Efficacy Scale (GSES) provides a measure of optimistic sense of personal competence [34].
- Cognitive flexibility: The Cognitive Flexibility Scale (CFS) measures a person’s awareness of communication alternatives, willingness to adapt to challenging situations and self-efficacy in being flexible [35].
- Quality of Life: The Quality of Life Enjoyment and Satisfaction Questionnaire Short Form (Q-LES-Q-SF) is a 16-item self-report measure of QoL [36]. Responses are scored on a 5-point Likert scale, with higher scores indicating greater enjoyment and satisfaction with life. The Q-LES-Q-SF measures enjoyment and satisfaction with overall well-being including physical health, mood, social and occupation functioning, relationships and daily functioning. The overall Q-LES-Q-SF score was used here.
- Disability: The Brief Disability Questionnaire (BDQ) is an assessment of overall perceived physical and mental disability [37].

### Statistical analyses

Given the lack of official criterion to classify EO- and LO-AN, we grouped the participants into three groups; EO-AN (AOO ≤ 14 years), TO-AN (AOO between 15 and 18 years) and LO-AN (AOO of above 18 years), as has been done previously [20, 24]. See Supplementary Material Table 3. and 4. for additional analyses using a median split.

Data analysis was performed using SPSS (IBM, SPSS Statistics Version 25). Means and standard deviations were calculated for continuous variables, and frequencies were measured for categorical variables. Given the cross-sectional design of the assessments, total frequencies that were collected for each measure are stated. For individuals with multiple assessments, only their first assessment data was used.

Between-group differences on continuous demographic variables were assessed using multiple one-way ANOVAs with Tukey post hoc analysis. Length of illness duration was used as a covariate measure in a series of ANCOVA analyses to determine the differences between AOO groups on measures of ED symptomatology, psychological distress and psychosocial function. Post hoc analysis was performed with a Bonferroni adjustment. Between-group comparisons of categorical variables were conducted using chi-squared tests of association; Fisher’s exact test was used to analyse categorical variables where cell counts were low (*n* < 5). For all analyses, significance was set at *p* < 0.05. Missing values were excluded on a list wise basis.

## Results

The mean age of the sample (*N =* 249, 94.4%F) was 27.04 ± 9.44 years at the time of assessment, of which 23.3% (*n* = 58) had an AOO of ≤14, 45.4% (*n* = 113) had an AOO between 15 and 18 years and 31.3% (*n* = 78) had an AOO of over 18. Participant characteristics and comparison between AOO groups are presented in Table 1.

**Table 1:** Participant characteristics

Measure	EO-AN M ± SD or N (%)	TO-AN M ± SD or N (%)	LO-AN M ± SD or N (%)	***p***-value
	*N* = 58	*N* = 113	*N* = 78	
Age	24.77 ± 7.94	24.96 ± 8.48	30.59 ± 10.06**	**<.001**
Age of onset	12.31 ± 2.35	16.19 ± 1.03**	24.01 ± 6.83**	**<.001**
Duration of illness	12.46 ± 8.94	8.08 ± 8.44*	6.39 ± 7.87**	**<.001**
Gender				0.832
Male	3 (5.2%)	5 (4.4%)	5 (6.4%)	
Female	55 (94.8%)	108 (95.6%)	73 (93.6%)	
Ethnicity				0.769
Caucasian	48 (82.8%)	89 (78.8%)	57 (73.1%)	
Other European	3 (5.2%)	5 (4.4%)	6 (7.7%)	
East Asian	2 (3.4%)	4 (3.5%)	1 (1.3%)	
Aboriginal and Torres Strait Islander	1 (1.7%)	2 (1.8%)	2 (2.6%)	
Other/Unknown	4 (6.9%)	13 (11.5%)	12 (15.4%)	
Education				0.177
Secondary School	24 (41.4%)	35 (31.0%)	16 (20.5%)	
Tertiary commenced/completed	30 (51.7%)	68 (60.2%)	50 (64.1%)	
Vocational	1 (1.7%)	3 (2.7%)	2 (2.6%)	
Other/Unknown	3 (5.2%)	7 (6.2%)	10 (12.8%)	
Employment				0.226
Student	19 (32.8%	48 (42.5%)	18 (23.1%)	
Full-time employed	4 (6.9%)	7 (6.2%)	9 (11.5%)	
Part-time employed	11 (19.0%)	23 (20.4%)	13 (16.7%)	
Home duties	1 (1.7%)	3 (2.7%)	4 (5.1%)	
Unemployed	4 (6.9%)	9 (8.0%)	7 (9.0%)	
Unable to work because of illness	17 (29.3%)	17 (15.0%)	23 (29.5%)	
Unknown/missing	2 (3.4%)	6 (5.3%)	4 (5.1%)	
Marital status				0.180
Never married	46 (79.3%)	88 (77.9%)	53 (67.9%)	
Widowed	–	–	1 (1.3%)	
Divorced/separated	1 (1.7%)	4 (3.5%)	8 (10.3%)	
Married/defacto	8 (13.8%)	10 (8.8%)	11 (14.1%)	
Unknown/missing	3 (5.2%)	11 (9.7%)	5 (6.4%)	

*compared to EO-AN, *p <* .01; **compared to EO-AN, *p <* .001

*EO-AN* early onset anorexia nervosa; *TO-AN* typical onset anorexia nervosa; *LO-AN* later onset anorexia nervosa

Individuals with EO-AN were significantly younger at assessment than those with LO-AN and had a significantly younger AOO and longer duration of illness than those with TO-AN or LO-AN. The comparison of ED symptomatology, psychological distress and psychosocial functioning between the three AOO groups (after adjustment for illness duration) are presented in Table 2.

**Table 2:** Comparison of ED severity, psychological distress and psychosocial function across AOO groups

Measure	EO-AN M ± SD or N (%)	TO-AN M ± SD or N (%)	LO-AN M ± SD or N (%)	***p***-value, effect size (η^**2**^)
	*N* = 58	*N* = 113	*N* = 78	
	*n* = 54	*n* = 107	*n* = 75	
BMI	17.07 ± 2.25	16.74 ± 2.27	16.75 ± 2.99	*p* = .463, η2 = .007
	*n* = 50	*n* = 82	*n* = 65	
AN Subtype				0.744
Restraint	45	75	61	
Binge-purge	5	7	4	
	*n* = 49	*n* = 85	*n* = 64	
EDE-Q				
Restraint	4.68 ± 1.37	3.88 ± 1.67	3.75 ± 1.82	*p* = .060, η2 = .030
Eating concern	4.28 ± 1.00	3.92 ± 1.36	3.44 ± 1.63*	*p* = **.031**, η2 = .037
Shape concern	5.26 ± 1.06	4.80 ± 1.22	4.29 ± 1.50**	*p* = **.004**, η2 = .057
Weight concern	4.91 ± 1.33	4.51 ± 1.38	3.92 ± 1.57*	*p* = **.019**, η2 = .042
Global	4.68 ± 1.21	4.18 ± 1.37	3.74 ± 1.54*	*p* = **.030**, η2 = .036
	*n* = 21	*n* = 40	*n* = 32	
Dysmorphic concern	13.33 ± 4.16	11.63 ± 4.87	10 ± 5.51*	*p* = **.029**, η2 = .077
	*n* = 55	*n* = 103	*n* = 74	
DASS-21				
Depression	28.76 ± 11.92	24.41 ± 11.76	22.81 ± 13.11	*p* = .087, η2 = .022
Anxiety	22.18 ± 11.01	18.52 ± 11.77	16.30 ± 10.85	*p* = .051, η2 = .026
Stress	28.55 ± 9.00	24.84 ± 10.02	23.46 ± 10.95	*p* = .116, η2 = .019
	*n* = 20	*n* = 41	*n* = 32	
Cognitive flexibility	40.85 ± 7.84	46.71 ± 7.76*	48.84 ± 8.67**	*p* = **.006**, η2 = .110
	*n* = 18	*n* = 39	*n* = 31	
Self-efficacy	23.33 ± 5.53	24.72 ± 5.38	26.42 ± 5.58	*p* = .210, η2 = .037
	*n* = 45	*n* = 81	*n* = 63	
Quality of life	34.07 ± 10.17	37.28 ± 10.05	37.16 ± 10.72	*p* = .530, η2 = .007
	*n* = 57	*n* = 106	*n* = 74	
Disability	12.65 ± 5.86	10.68 ± 5.07	10.69 ± 5.68	*p* = .117, η2 = .019

*compared to EO-AN, *p <* .05; **compared to EO-AN, *p <* .005

*EO-AN* Early onset anorexia nervosa; *TO-AN* Typical onset anorexia nervosa; *LO-AN* Later onset anorexia nervosa; *BMI* Body mass index; *EDE-Q* Eating disorder examination questionnaire; *DASS-21* Depression anxiety stress scale

The EO-AN group demonstrated significantly higher levels of ED symptoms in subscales of eating concern, shape concern, weight concern and global score of the EDE-Q, compared to the LO-AN group. The EO-AN group also reported significantly higher levels of dysmorphic concern than the LO-AN group. Individuals with EO-AN had significantly lower scores of cognitive flexibility, compared to the TO-AN and LO-AN groups. There were no significant differences between groups on other variables.

## Discussion

The current study utilised a large sample of treatment-seeking adults at an out-patient ED service to investigate ED symptomatology, psychological distress and psychosocial function between EO-AN, TO-AN and LO-AN patients. Our hypotheses that those with EO-AN would demonstrate increased ED symptomatology, psychological distress and more impaired psychosocial function than those with TO-AN or LO-AN were partly supported. There were no differences in BMI or AN subtype between the three groups. However, the EO-AN group reported a significantly longer illness duration than both TO-AN and LO-AN groups. After controlling for the impact of illness duration, the EO-AN group reported significantly increased ED symptomatology and dysmorphic concern than those with LO-AN. Moreover, the EO-AN group demonstrated significantly decreased cognitive flexibility as compared to both the TO-AN and LO-AN groups. There were no differences between groups on psychological distress or other psychosocial outcomes.

In accordance with prior research, our observations that patients with EO-AN reported a longer illness duration, higher ED symptomatology and dysmorphic concern than those with LO-AN, which indicates that EO-AN may present with a more severe form of illness [25, 38]. The increased severity in ED symptomatology demonstrated by the EO-AN group was consistent across all domains of the EDE-Q aside from restraint, which demonstrated a trend towards being increased in the EO-AN group. This supports previous findings of more severe ED behaviours, such as complete refusal of oral intake and more severe restriction, in patients with EO-AN. Similarly, distorted self-perception of body image, a key feature of AN [29, 39], was significantly higher in those with EO-AN compared to LO-AN, consistent with previous findings [25]. Contributing factors to the increased ED psychopathology seen in the EO-AN group may include various biological and environmental factors, which differ across development. These include pressures experienced by younger patients, such as changes related to puberty and increased susceptibility to external negative influences on body image perception and idealisation [40]. It has been postulated that onset of AN prior to puberty may intensify perceived body image ideals [41], whereby the associated increase in adipose tissue and widening of the hips in adolescent females during puberty may exacerbate cognitions related to thin body ideal [18]. Moreover, experience of body change in the EO-AN group may also be substantially influenced by social media ideals, peer relationships and the emergence of gender roles [42, 43]. The importance of physical attractiveness and the consolidation of sexuality have been demonstrated to influence self-concept and psychological profile at this stage of development [44]. Moreover, rates of teasing and bullying have been demonstrated to be higher in EO-AN than LO-AN [21], supportive of the theory that early developmental trauma may contribute to increased levels of psychological distress as well as enduring patterns of body image disturbance [45, 46].

Another feature that is widely associated with AN is cognitive inflexibility [47, 48], which involves deficits in the ability to adapt thinking or attention to shifting goals or environmental stimuli [49]. The current study demonstrated significantly lower levels of cognitive flexibility in patients with EO-AN compared to both TO-AN and LO-AN, supportive of previous findings of lower metacognitive abilities in patients with EO-AN [50]. Decreased cognitive flexibility, as demonstrated in the EO-AN group, may manifest in heightened rigidity in thinking and be reflected in more severe ED cognitions [51], resulting in behaviours such as categorisation of food and calorie counting [52]. Moreover, cognitive inflexibility may also lead to problems in finding solutions to managing difficulties and distress, therefore maintaining maladaptive thoughts and behaviours in AN [52], contributing to the challenges faced in psychotherapeutic interventions in this patient group. Specifically, diminished cognitive flexibility may be a limiting factor in cognitive behaviour therapy interventions, whereby a lack of communication of alternatives may lead to poor engagement with treatment and suboptimal outcomes of therapy. Indeed, cognitive inflexibility and obsessional thinking have been shown to predate the onset of AN, persist over the course of the illness and contribute to later relapses in adulthood [53, 54].

Investigations into psychological distress in the current study found no significant differences between the three AOO groups in measures of depression, anxiety and stress. However, there was a trend for increased anxiety in individuals with EO-AN compared to TO-AN and LO-AN, which may be due to the abovementioned developmental and environmental influences experienced by this group of patients. It has also been suggested that EO-AN is under stronger influence of biological processes such as pre-illness alterations in neural circuits [55], which may lead to higher expression of distress and anxiety symptoms. Psychological distress was universally high across all three groups, which is representative of the established high rates of comorbid anxiety and depressive disorders across varying AOO groups in AN [24, 38, 56, 57].

## Conclusion

The current study builds upon previous research and has demonstrated that a large community-based treatment-seeking sample of patients with EO-AN exhibit more severe ED pathology and higher dysmorphic concern, compared with LO-AN patients, which are not attributable to a longer illness duration. Moreover, individuals with EO-AN demonstrated decreased cognitive flexibility compared to those with TO-AN or LO-AN. The disparities between AOO groups have potential implications for prognostic and treatment outcomes. Indeed, knowledge of increased ED severity and decreased cognitive flexibility may enable clinicians to adopt more tailored interventions for this vulnerable group. Further investigation into understanding the early developmental influences on illness manifestation could highlight unique targets of future interventions.

The limitations of the current study include the cross-sectional nature of assessments, with not all outcomes completed by the participants and a lack of long-term follow up. Other limitations include the inclusion of self-reported AOO, measures of ED symptomatology and psychological distress. Future research should investigate the long-term implications of AOO on treatment outcomes. This will enable informed early detection and intervention with EO patients as well as targeted interventions.

## Supplementary information

**Additional file 1.** Results from a data-driven approach (median split) to dichotomise the sample into two AOO groups.

## Data Availability

The datasets used and/or analysed during the current study are available from the corresponding author on reasonable request.

## Abbreviations

AN: Anorexia nervosa
EO: Early onset
TO: Typical onset
LO: Later onset
ED: Eating disorder
AOO: Age of onset
BMI: Body mass index
BETRS: Body image and eating disorders recovery service
DSM-5: Diagnostic and statistical manual of mental disorders, 5th edition
EDE-Q: Eating disorder examination questionnaire
DCQ: Dysmorphic concern questionnaire
DASS-21: Depression anxiety stress scale
GSES: General self-efficacy scale
CFS: Cognitive flexibility scale
Q-LES-Q-SF: Quality of life enjoyment and satisfaction questionnaire short form
BDQ: Brief disability questionnaire
SPSS: Statistical package for the social sciences
