# Supplementary material for: A comparison of eating disorder symptomatology, psychological distress and psychosocial function between early, typical and later onset anorexia nervosa
Source: J Eat Disord. 2020 Nov 4;8:56. doi: 10.1186/s40337-020-00337-w (PMC7640671; doi:10.1186/s40337-020-00337-w)
Supplement: Supplementary file 1 — Additional file 1. Results from a data-driven approach (median split) to dichotomise the sample into two AOO groups. [file 40337_2020_337_MOESM1_ESM.docx]

**Supplementary Material**

These additional analyses detail the results from a data-driven approach to dichotomise the sample into AOO groups. A median split of AOO (median AOO=16) was conducted to group the patients into EO-AN (AOO of ≤ 16) or LO-AN (AOO of ED of > 16).

Table 3. Participant characteristics

| **Measure** | **All AN**  **M ± SD or N (%)**  ***N=*269** | **EO-AN**  **M ± SD or N (%)**  ***N=*128** | **LO-AN**  **M ± SD**  ***N=*121** | ***p*-value*** |
| --- | --- | --- | --- | --- |
| Age at assessment | 27.04 ± 9.44 | 25.09 ± 8.68 | 28.35 ± 9.54 | **0.005** |
| Age of onset | 17.74 ± 6.05 | 14.05 ± 2.27 | 21.64 ± 6.35 | **<.001** |
| Duration of illness (years) | 8.86 ± 9.00 | 10.71 ± 9.20 | 6.30 ± 2.73 | **<.001** |
| Gender |  |  |  | 0.856 |
| Male | 15 (5.6%) | 7 (5.5%) | 6 (5.0%) |  |
| Female | 254 (94.4%) | 121 (94.5%) | 115 (95.0%) |  |
| Ethnicity |  |  |  | 0.624 |
| Aboriginal & Torres Strait Islander | 5 (1.9%) | 2 (1.6%) | 3 (2.5%) |  |
| Caucasian | 209 (77.7%) | 106 (82.8%) | 88 (72.7%) |  |
| Asian | 8 (3.0%) | 4 (3.1%) | 3 (2.5%) |  |
| Other European | 16 (5.9%) | 5 (3.9%) | 9 (7.4%) |  |
| Other | 6 (2.2%) | 2 (1.6%) | 3 (2.5%) |  |
| Unknown/missing | 25 (9.3%) | 9 (7.0%) | 15 (12.4%) |  |
| Education |  |  |  | **0.001** |
| Secondary | 79 (29.4%) | 52 (40.7%) | 23 (19.0%) |  |
| Tertiary commenced/completed | 161 (59.9%) | 67 (52.4%) | 81 (67.0%) |  |
| Vocational | 7 (2.6%) | 3 (2.3%) | 3 (2.5%) |  |
| Unknown/missing | 22 (8.1%) | 6 (4.7%) | 14 (11.6%) |  |
| Employment |  |  |  | 0.465 |
| Student | 90 (33.5%) | 46 (35.9%) | 39 (32.2%) |  |
| Full-time employed | 22 (8.2%) | 7 (5.5%) | 13 (10.7%) |  |
| Part-time employed | 49 (18.2%) | 29 (22.7%) | 18 (14.9%) |  |
| Home duties | 10 (3.7%) | 4 (3.1%) | 4 (3.3%) |  |
| Unemployed | 24 (8.9%) | 9 (7.0%) | 11 (9.1%) |  |
| Unable to work because of illness | 60 (22.3%) | 28 (21.9%) | 29 (24.0%) |  |
| Unknown/missing | 14 (5.2%) | 2 (1.6%) | 7 (5.8%) |  |
| Marital Status |  |  |  | 0.236 |
| Never married | 198 (73.6%) | 98 (76.6%) | 89 (73.6%) |  |
| Widowed | 2 (0.7%) | - | 1 (0.8%) |  |
| Divorced/separated | 14 (5.2%) | 4 (3.1%) | 9 (7.5%) |  |
| Married/defacto | 33 (12.3%) | 15 (11.7%) | 14 (11.6%) |  |
| Unknown/missing | 22 (8.2%) | 11 (8.6%) | 8 (6.6%) |  |

AN: anorexia nervosa; M: mean; SD: standard deviation; EO-AN: early-onset anorexia nervosa; LO-AN: later onset anorexia nervosa; *comparison of EO-AN and LO-AN groups.

Table 4. Comparison of ED severity, psychological distress and psychosocial function between EO-AN and LO-AN.

| **Measure** | **EO-AN** | **LO-AN** | ***p*-value** |
| --- | --- | --- | --- |
|  | **M ± SD** | **M ± SD** |  |
|  | ***N=*128** | ***N=*121** |  |
|  | n=122 | n=119 |  |
| BMI | 16.94 ± 2.28 | 16.70 ± 2.73 | 0.461 |
|  | n = 99 | n = 94 |  |
| EDE-Q |  |  |  |
| Restraint | 4.32 ± 1.58 | 3.74 ± 1.75 | **0.017** |
| Eating Concern | 4.07 ± 1.17 | 3.63 ± 1.59 | **0.030** |
| Shape Concern | 5.02 ± 1.11 | 4.47 ± 1.48 | **0.004** |
| Weight Concern | 4.72 ± 1.30 | 4.10 ± 1.58 | **0.003** |
| Global Score | 4.44 ± 1.25 | 3.87 ± 1.55 | **0.005** |
|  | n = 48 | n=45 |  |
| Dsymorphic concern | 12.63 ± 4.42 | 10.20 ± 5.43 | **0.020** |
| AN subtype | n=99 | n=98 | 0.191 |
| Restraint | 88 (91.0%) | 93 (90.0%) |  |
| Binge-purge | 11 (8.0%) | 5 (8.0%) |  |
| DASS-21 | n=117 | n=115 |  |
| Depression | 26.43 ± 11.71 | 23.41 ± 12.93 | 0.064 |
| Anxiety | 20.38 ± 11.07 | 16.96 ± 11.65 | **0.023** |
| Stress | 26.97 ± 8.93 | 23.55 ± 11.19 | **0.011** |
|  | n = 45 | n = 43 |  |
| Self-efficacy | 24.70 ± 5.20 | 25.36 ± 5.88 | 0.580 |
|  | n=47 | n=46 |  |
| Cognitive flexibility | 44.40 ± 8.38 | 48.00 ± 8.41 | **0.042** |
|  | n = 98 | n = 91 |  |
| Quality of life | 36.01 ± 9.83 | 36.98 ± 10.89 | 0.522 |
|  | n=122 | n=115 |  |
| Disability | 11.57 ± 5.44 | 10.72 ± 5.56 | 0.239 |

AN: anorexia nervosa; M: mean; SD: standard deviation; EO-AN: early-onset anorexia nervosa; LO-AN: later onset anorexia nervosa; BMI: body mass index; EDE-Q: eating disorder examination questionnaire.
